# Prognostic significance of lymphovascular invasion in patients with pT1b esophageal squamous cell carcinoma

**DOI:** 10.1186/s12885-023-10858-7

**Published:** 2023-04-22

**Authors:** Linxiu Liu, Hua Lin, Guihua Shen, Yong Liu, Xiumin Qin, Yanling Yuan, Bingzhi Wang, Liyan Xue

**Affiliations:** 1 Department of Pathology, National Cancer Center/National Clinical Research Center for Cancer/Cancer Hospital, Chinese Academy of Medical Sciences and Peking Union Medical College, Beijing, China; 2 Department of Medical Record, National Cancer Center/National Clinical Research Center for Cancer/Cancer Hospital, Chinese Academy of Medical Sciences and Peking Union Medical College, Beijing, 100021 China; 3 Department of Endoscopy, National Cancer Center/National Clinical Research Center for Cancer/Cancer Hospital, Chinese Academy of Medical Sciences and Peking Union Medical College, Beijing, China

**Keywords:** Esophageal squamous cell carcinoma, Lymphovascular invasion, Lymph node metastasis, Recurrence-free survival, Overall survival, Distant metastasis-free survival

## Abstract

**Background:**

Lymphovascular invasion (LVI) is a crucial predictor of lymph node metastasis (LNM). However, few studies have investigated the LVI positivity rate and its clinical significance in pT1b esophageal squamous cell carcinoma (ESCC) using immunohistochemistry and elastin staining.

**Methods:**

We collected data from158 patients with pT1b ESCC who had undergone radical esophagectomy. All paraffin blocks of invasive carcinoma from each patient were subjected to HE staining, elastin staining + CK (AE1/AE3) immunohistochemistry (E&IHC), and CD31/D2-40 + CK (AE1/AE3) double immunohistochemistry (D-IHC). The LVI was classified into types, i.e., vascular invasion (VI) and lymphatic vessel invasion (LI), and its location, quantity, and clinical significance were explored.

**Results:**

The positivity rates of VI by E&IHC (E-VI), VI by CD31D-IHC (CD31-VI), and LI by D2-40 D-IHC (D2-40-LI) were significantly higher than those obtained by HE staining (*P* < 0.001, respectively). CD31-VI and E-VI were independent adverse prognostic factors for recurrence-free survival (RFS), and they were significantly associated with poor distant metastasis-free survival and overall survival in pT1b ESCC. Intratumoral LVI was also crucial in pT1b ESCC, and L2 (the count of D2-40-LI was 5 or more) was the strongest predictor for LNM and RFS in pT1b ESCC.

**Conclusion:**

E&IHC and D-IHC can dramatically improve the detection rate of LVI in pT1b ESCC, and the classification and grading of LVI can help to improve the prediction of LNM and prognosis.

**Supplementary Information:**

The online version contains supplementary material available at 10.1186/s12885-023-10858-7.

## Background

Esophageal squamous cell carcinoma (ESCC) is one of the most common malignant tumors worldwide, particularly in East Asia. Superficial ESCC (SESCC) is defined as a tumor restricted to the mucosa or submucosa, irrespective of regional lymph node metastasis (LNM), and can be classified as either pT1a or pT1b. The extent of tumor invasion in pT1a is the lamina propria or muscularis mucosa, while tumor invasion in pT1b is restricted to the submucosa [1]. The frequency of LNM is correlated with the depth of invasion, and many studies have reported the occurrence of LNM in SESCC [2–4]. The incidence of LNM in patients with pT1a ESCC is 1.3–8.1%, but it is higher in patients with pT1b ESCC, ranging from 23.3 to 51.2% [5–10]. Compared with pT1a ESCC patients, pT1b ESCC patients have a much higher risk of LNM and recurrence. However, most studies have focused on SESCC (pT1a and pT1b), and few studies have been conducted on the pT1b stage alone.pT1b ESCC patients without LNM only need surveillance, but patients with synchronous or heterochronous LNM may need radiotherapy, chemotherapy, or immunotherapy. Therefore, how to more accurately predict the possibility of LNM and risk of recurrence using pathological features is critical for determining the optimal treatment for patients with pT1b ESCC.

Lymphovascular invasion (LVI) is deemed an essential step in tumor metastasis and a significant predictor of metastasis. Many studies have shown that LVI is important for LNM in SESCC [11–13]. However, the reported frequency of LVI in SESCC varies widely, ranging from 6.2% to 60% [11, 13–20]. In most studies, LVI was recognized only on hematoxylin and eosin (HE) staining, resulting in inaccurate findings and subjective differences. In early-stage colorectal cancer, double immunohistochemistry (D-IHC) can enhance the detection of LVI, but a lack of relevant research exists in ESCC [21]. Additionally, Castonguay M C et al. showed that the identification of vascular invasion is greatly enhanced by elastin staining and is associated with various adverse clinicopathological features [22]. Only a few studies have used both elastin staining and IHC to evaluate LVI [23, 24], but no study has used elastin staining + CK (AE1/AE3) immunohistochemistry (E&IHC) and D-IHC in ESCC at the T1b stage.

On the other hand, most studies on LVI in ESCC have not attempted to differentiate between vascular invasion (VI) and lymphatic vessel invasion (LI) [13–16]. However, in patients with bladder cancer, VI has a stronger association with recurrence and poor survival than LI, and VI has been shown to be associated with more widespread metastases to distant organs [25]. Similarly, other scholars have shown that the presence of vascular invasion (but not lymphatic invasion) could be considered an indicator of high biological aggressiveness and may be a decisive prognostic factor in patients with colorectal cancer [26]. Therefore, it is necessary to study VI and LI separately. The location of LVI should also be a concern. In breast cancer research, only peritumoral LVI is of value [27]. Mori D et al*.* demonstrated that intratumoral LI was not related to LNM. Only LI of the intramucosal and submucosal peritumoral areas was significantly associated with LNM in ESCC patients [28]. In addition, grouping hepatocellular carcinoma patients based on the microvascular invasion count (> 5 and ≤ 5) helped to determine the risk of recurrence and prognosis [29], and the grading index has been included as a factor in the TNM staging system.

Therefore, in the present investigation, all paraffin blocks containing infiltrating carcinoma from each patient were subjected to HE staining, E&IHC, and D-IHC to explore the actual LVI positivity rate in patients with pT1b ESCC. Additionally, we refined studies on LVI by focusing on its location and quantity and explored its clinical significance.

## Methods

### Patient selection

One hundred fifty-eight patients with pT1b ESCC who underwent radical surgical resection using thoraco-abdominal lymphadenectomy at the National Cancer Center/National Clinical Research Center for Cancer/Cancer Hospital, Chinese Academy of Medical Sciences and Peking Union Medical College between February 1990 and January 2004 and had complete pathological and clinical data were evaluated. None of the patients received radiotherapy, chemotherapy, or biological therapy before surgery. The study protocol was approved by the Institutional Review Board of the National Cancer Center/National Clinical Research Center for Cancer/Cancer Hospital, Chinese Academy of Medical Sciences and Peking Union Medical College (Approval No.NCC2018AA-036). All procedures were conducted in accordance with the ethical standards of the Declaration of Helsinki.

### E&IHC and D-IHC

The tumor lesions in each case were fully sampled for microscopic examination. We selected all paraffin blocks containing infiltrating carcinoma from each patient and then made serial 4 μm slices for HE staining, E&IHC and D-IHC.

E&IHC was performed as follows. The slices were dewaxed, antigen retrieved, and subjected to immunohistochemistry with CK (AE1/AE3) (mouse anti-human monoclonal antibody; Dako, Carpinteria, CA). Next, improved aldehyde complex red staining was performed to detect venous elastic fiber [30]. The elastic fiber appeared blue and purple, and tumor cells appeared brown.

For D-IHC, sequential double immunohistochemical staining was performed using a BOND III Immunostainer (Leica Microsystems) and a Bond Polymer Refine Detection Kit (DS9800) as the first stain and a Bond Polymer Refine Red Detection Kit (DS9390) as the second stain. D2-40 and CD31 (mouse anti-human monoclonal antibody; Beijing Zhong Shan Golden Bridge Biotech Co., Ltd.) were visualized with DAB chromogen, and CK (AE1/AE3) (mouse anti-human monoclonal antibody; Dako, Carpinteria, CA) was visualized with Fast Red chromogen.

Normal esophageal tissue was used for each batch of immunohistochemical staining to establish one external positive control and one negative control. In the negative control, buffers were used instead of primary antibodies. In addition, negative and positive internal controls are also present in each section.

### Interpretation of LVI

**Fig. 1:**
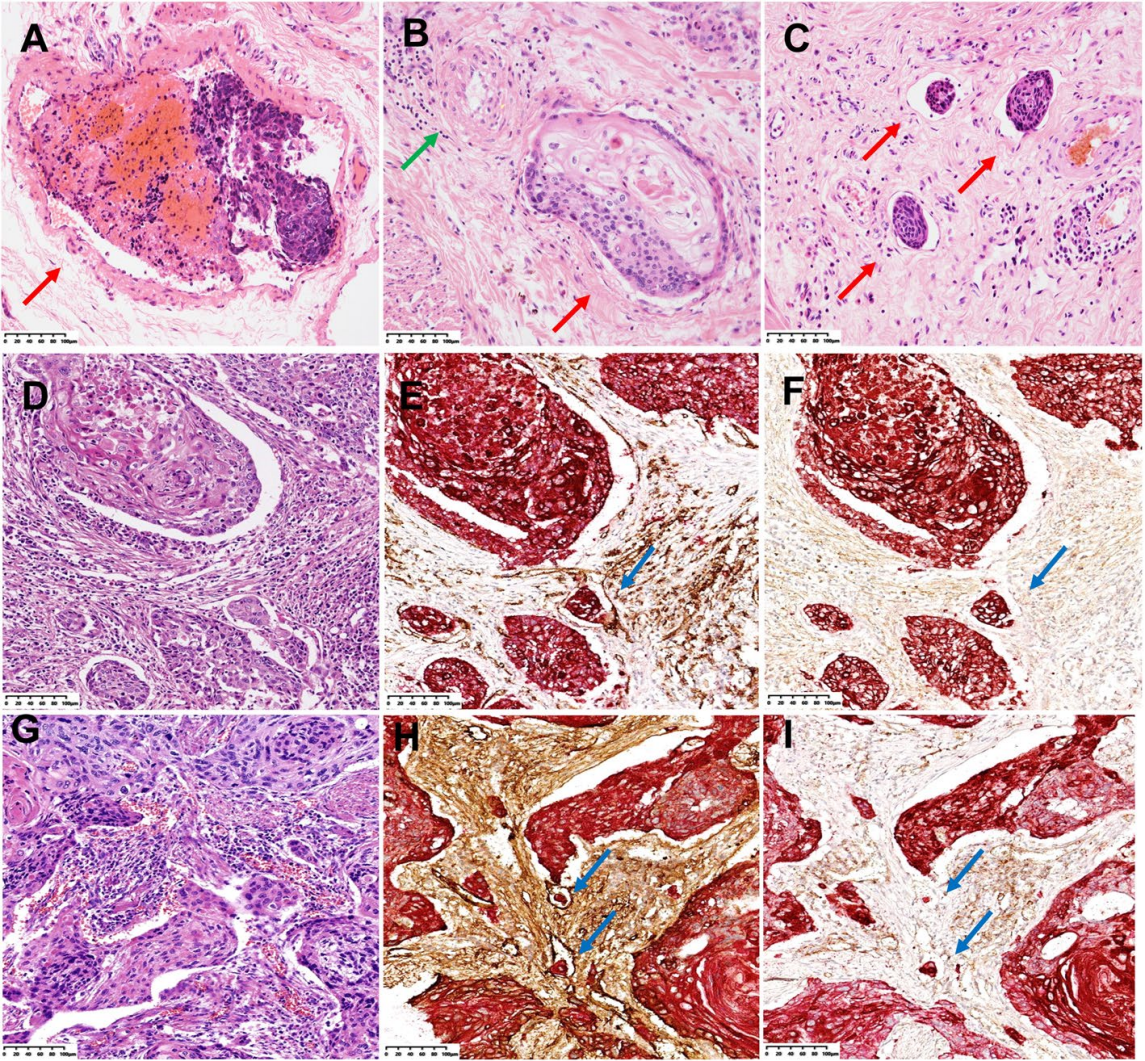
Determination of lymphovascular invasion (LVI) by hematoxylin and eosin (HE) staining and double immunohistochemistry (D-IHC) **A** Vascular invasion (red arrow, HE). **B** Venous invasion (red arrow) adjacent to an isolated artery (green arrow) (HE). **C** Lymphatic vessel invasion (red arrow, HE). **D** Lymphovascular invasion is indefinite (HE). **E** Vascular invasion (CD31 + CK (AE1/AE3) D-IHC). The blue arrow shows the presence of tumor cells (red color) in the lumen with endothelial cells positive (brown color) for CD31. **F** Vascular invasion (D2-40 + CK (AE1/AE3) D-IHC). The endothelial cells of the vessel are negative (blue arrow) for D2-40. **G** Lymphovascular invasion is indefinite (HE). **H** Lymphatic vessel invasion (D2-40 + CK (AE1/AE3) D-IHC). The blue arrow shows the presence of tumor cells (red color) in the lumen with endothelial cells positive (brown color) for D2-40. **I** Lymphatic vessel invasion (CD31 + CK (AE1/AE3) D-IHC). The endothelial cells of the vessel are negative (blue arrow) for CD31. All images are magnified 200 ×

**Fig. 2:**
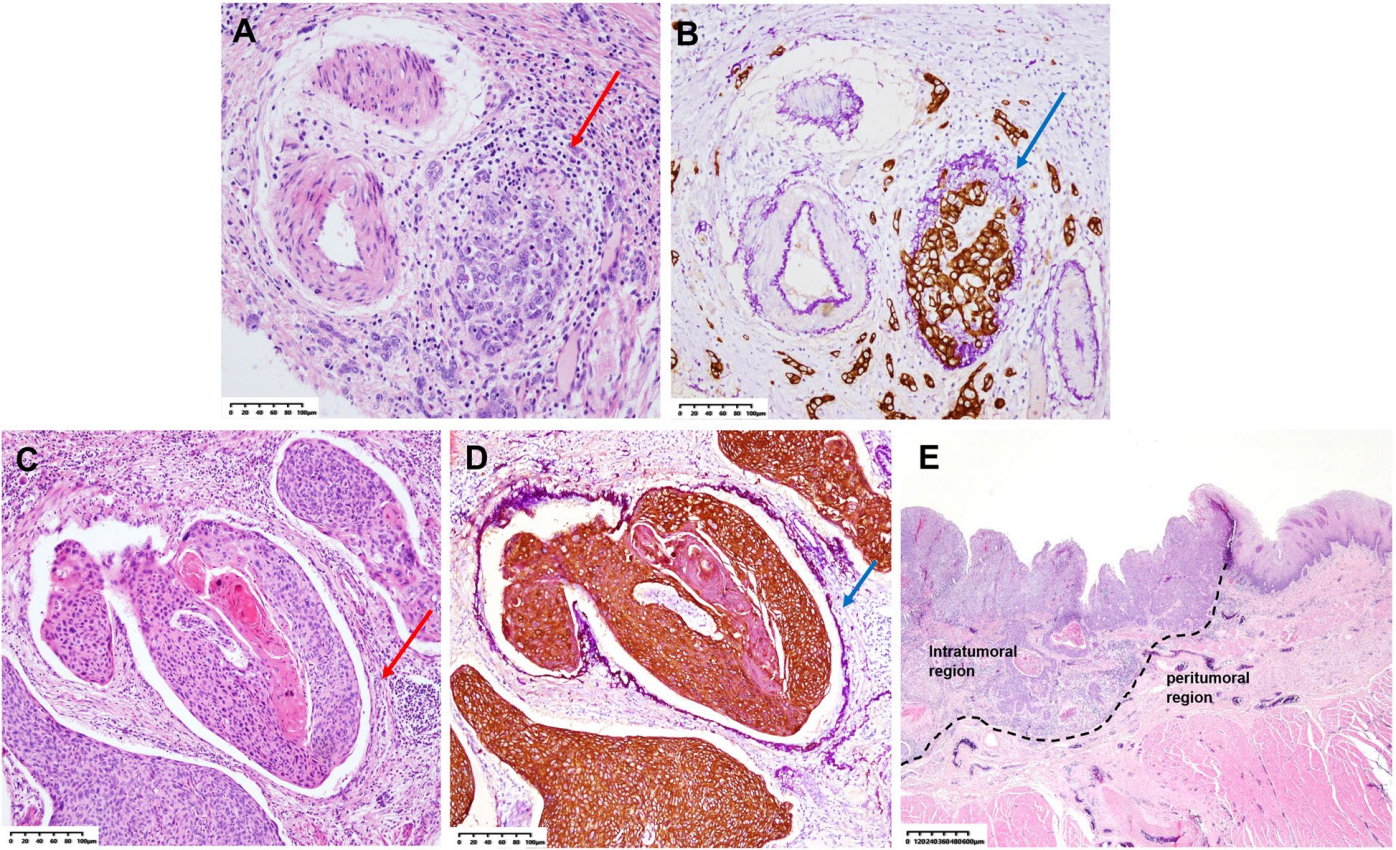
Determination of vascular invasion by elastin staining + CK (AE1/AE3) immunohistochemistry (E&IHC) and determination of the intratumoral region and peritumoral region. **A** Vascular invasion is indefinite (HE). The red arrow shows carcinoma nest adjacent to the artery, suggestive of vascular invasion. **B** Vascular invasion (E&IHC). The blue arrow shows the elastic fiber (purple color) clearly outlining the structure of the blood vessel with carcinoma nest (brown color) invasion. **C** Vascular invasion is indefinite (HE). The red arrow shows a space between the carcinoma nest and the surrounding fibrous stroma, suspected to be caused by cancer nest contraction. **D** Vascular invasion (E&IHC). The blue arrow shows the elastic fiber (purple color) clearly outlining the structure of the blood vessel with carcinoma nest (brown color) invasion, which is identified as vascular invasion. **E** Determination of the intratumoral region and peritumoral region (HE). The peritumoral region included the junction between the tumor and normal tissue and the normal tissue area outside the tumor (including the region at the dotted line and the region from the dotted line to the esophageal fibrous membrane). The images of (**A**), (**B**), (**C**), and (**D**) are magnified 200 × , and (**E**) is 25 ×

The results were independently interpreted by two pathologists (LL and BW). A multi-head microscope was used to reach a consensus in the case of inconsistent results. The presence of tumor cells in the lumen with endothelial cells with a gap between the tumor cells and vessel wall was judged as LVI. The presence of red blood cells in the lumen or smooth muscle around the vessel by HE staining was judged as VI and recorded as HE-VI (Fig. 1A). The presence of tumor cell nests adjacent to isolated arteries (no accompanying veins) by HE staining was also recorded as HE-VI (Fig. 1B). The absence of red blood cells in the lumen and without smooth muscle around the vessel was judged as LI and recorded as HE–LI (Fig. 1 ). The presence of cancer cells in the lumen with endothelial cells by D-IHC was judged as LVI. LVI with endothelial cells positive for CD31 and negative for D2-40 (Fig. 1D/E/F) was recorded as CD31-VI. LVI with endothelial cells strongly positive for D2-40 and negative or weakly positive for CD31 was recorded as D2-40-LI (Fig. 1G/H/I). VI (shown by E&IHC) was defined as tumor cells in the lumen surrounded by elastic fiber with at least 2/3 integrity and was recorded as E-VI (Fig. 2A/B/C/D). The locations of VI and LI in the intratumoral region or peritumoral region (including the normal tissue area outside the tumor and the junction between the tumor and normal tissue) were recorded(Fig. 2E). The quantities of VI and LI were also recorded.

### Other clinicopathological variables and follow-up

We recorded the tumor location (upper thoracic, middle thoracic, or lower thoracic), macroscopic type (erosive type, papillary type, plaque-like type, ulcerative type, or intraluminal mass type) [10], depth of tumor invasion (sm1, sm2 or sm3) [31] and degree of differentiation (well, moderately, poorly differentiated, basaloid squamous cell carcinoma or spindle cell/sarcomatoid squamous cell carcinoma) [1]. We also measured tumor thickness and submucosal invasion thickness. According to our previous studies, the cutoff points of tumor thickness and submucosal invasion thickness were 3000 μm and 2000 μm, respectively [32]. The number of lymph nodes dissected and whether lymph nodes had metastasized were recorded. Regarding follow-up information, we collected the time to recurrence, distant metastasis, or death and then calculated recurrence-free survival (RFS), distant metastasis-free survival (DMFS), and overall survival (OS).

### Statistical analysis

The data were statistically processed using IBM SPSS Statistics for Windows, version 25.0 (IBM Corp., Armonk, NY, USA). The relationship between the clinicopathological variables and LNM was analyzed by logistic regression analysis. Receiver operating characteristic (ROC) curves were used to determine the cutoff points of counting variables, and the Kaplan–Meier method and log-rank test were used to analyze survival. The significance of clinicopathological markers relative to RFS, DMFS, and OS were analyzed using a Cox proportional hazards regression model (univariate analysis and multivariate analysis). *P* < 0.05 was regarded as statistically significant.

## Results

Three hundred sixty-two paraffin blocks containing invasive carcinoma (1–6 paraffin blocks and an average of 2.3 blocks per patient) were selected. The positivity rates of HE-VI and HE–LI were 4.4% (7/158) and 22.2% (35/158), respectively. The positivity rates of E-VI, CD31-VI, and D2-40-LI were 69% (109/158), 25.3% (40/158), and 49.4% (78/158), respectively, which were higher than those of HE-VI and HE–LI (*P* < 0.001, respectively). Compared with HE staining, E&IHC and CD31 D-IHC increased the positivity rate of VI by 64.6% and 20.9%, respectively, and D2-40 D-IHC increased the detection rate of LI by 27%.

Synchronous LNM was present in 45 of 158 patients. No significant correlation was found between VI and LNM, regardless of the staining method used (Table 1). HE–LI was significantly correlated with LNM (*P* = 0.001). D2-40-LI showed no significant correlation with LNM (*P* = 0.094). However, peritumoral D2-40-LI was significantly correlated with LNM (*P* = 0.015). The count of D2-40-LI of all paraffin blocks was 1 to 73 in the positive case of LI. A ROC curve was drawn to obtain the optimal cutoff point of the D2-40-LI count for predicting LNM, which was 4.5 (area under the curve = 0.633, and Youden index = 0.285). According to the D2-40-LI count of all paraffin blocks, D2-40-LI was subdivided into three groups, L0 (0), L1 (1–4), and L2 (≥ 5). There was a statistically significant difference between L2 and L0 for LNM but not between L1 and L0 (Table 1). And the Odds ratio of L2 (6.889) for LNM was greater than that of HE–LI (3.765). For other clinicopathological indicators, univariate regression analysis showed that poor differentiation was a poor prognostic factor for LNM (Table 1). Multivariate logistic regression analysis revealed that L2 was the strongest independent predictor of LNM, with an odds ratio of 9.023 (95% CI: 3.026–26.899; *P* < 0.001; Table 2).

**Table 1:** Relationship between clinicopathological characteristics and lymph node metastasis (LNM) in 158 pT1b ESCC^a^ patients

Clinicopathological characteristics	Total	Invasion depth			LNM		Univariate logistic regression		
		sm1	sm2	sm3	No (%)	Yes (%)	Odds ratio	95% CI	*P*
Lymphovascular invasion									
HE staining									
HE-VI^b^ +	7	0	3	4	5 (71.4)	2 (28.6)	1.005	0.188–5.377	0.996
HE-VI-	151	15	33	103	108 (71.5)	43 (28.5)	1		
HE–LI^c^ +	35	4	5	26	17 (48.6)	18 (51.4)	3.765	1.711–8.282	0.001
HE–LI-	123	11	31	81	96 (78)	27 (22)	1		
Elastin staining + CK (AE1/AE3) IHC ^d^									
E-VI^e^ +	109	4	24	81	74 (67.9)	35 (32.1)	1.845	0.827–4.116	0.135
E-VI-	49	11	12	26	39 (79.6)	10 (20.4)	1		
Peritumoral E-VI +	62	4	15	43	41 (66.1)	21 (33.9)	1.537	0.763–3.094	0.229
Peritumoral E-VI-	96	11	21	64	72 (75.0)	24 (25.0)	1		
CD31 + CK (AE1/AE3) D-IHC ^f^									
CD31-VI^g^ +	40	1	8	31	25 (62.5)	15 (37.5)	1.760	0.821–3.773	0.146
CD31-VI-	118	14	28	76	88 (74.6)	30 (25.4)	1		
Peritumoral CD31-VI +	32	1	6	25	21 (65.6)	11 (34.4)	1.417	0.619–3.247	0.409
Peritumoral CD31-VI-	126	14	30	82	92 (73)	34 (27)	1		
D2-40 + CK (AE1/AE3) D-IHC									
D2-40-LI^h^ +	78	6	16	56	51 (65.4)	27 (34.6)	1.824	0.904–3.680	0.094
D2-40-LI-	80	9	20	51	62 (77.5)	18 (22.5)	1		
Peritumoral D2-40-LI +	67	6	16	47	41 (61.2)	26 (38.8)	2.403	1.187–4.863	0.015
Peritumoral D2-40-LI-	91	9	22	60	72 (79.9)	19 (20.1)	1		
L0 ^i^	80	9	20	51	62(77.5)	18 (22.5)	1		
L1	50	3	8	39	39 (78.0)	11 (22.0)	0.881	0.379–2.051	0.947
L2	28	3	8	17	12 (42.9)	16 (57.1)	6.889	2.540–18.685	< 0.001
Age									
≥ 60	58	7	19	32	42 (72.4)	16 (27.6)	0.933	0.454–1.916	0.849
< 60	100	8	17	75	71 (71.0)	29 (29.0)	1		
Sex									
Male	43	5	9	29	33 (76.7)	10 (23.3)	0.693	0.308–1.559	0.375
Female	115	10	27	78	80 (69.6)	35 (30.4)	1		
Degree of differentiation									
Well	31	2	6	23	27 (87.1)	4 (12.9)	1		
Moderate	60	7	18	35	42 (70.0)	18 (30.0)	2.893	0.883–9.475	0.079
Poor	40	3	7	30	25 (62.5)	15 (37.5)	4.050	1.184–13.853	0.026
Basaloid	19	1	5	13	14 (73.7)	5 (26.3)	2.411	0.557–10.429	0.239
Sarcomatoid	8	2	0	6	5 (62.5)	3 (37.5)	4.050	0.686–23.901	0.123
Tumor location									
Upper thoracic	31	1	10	20	24 (77.4)	7 (22.6)	0.781	0.303–2.015	0.609
Middle thoracic	103	14	20	69	75 (72.8)	28 (27.2)	1		
Lower thoracic	24	0	6	18	14 (58.3)	10 (41.7)	1.913	0.762–4.802	0.167
Macroscopic type									
Plaque-like	85	8	15	62	59 (69.4)	26 (30.6)	1		
papillary	18	2	5	11	16 (88.9)	2 (11.1)	0.284	0.061–1.324	0.109
Erosive	31	2	14	15	23 (74.2)	8 (25.8)	0.789	0.3121–1.995	0.617
Ulcerative	9	1	1	7	5 (55.6)	4 (44.4)	1.185	0.451–7.313	0.402
Intraluminal mass	15	2	1	12	10 (66.7)	5 (33.3)	1.135	0.353–3.650	0.832
Depth of tumor invasion ^j^									
sm1	15	-			12 (80.0)	3 (20.0)	1		
sm2	36	-			28 (77.8)	8 (22.2)	1.143	0.258–5.067	0.861
sm3	107	-			73 (68.2)	34 (31.8)	1.863	0.493–7.037	0.359
Tumor thickness									
≥ 3000 μm	125	8	20	97	85 (68.0)	40 (32.0)	2.635	0.947–7.331	0.063
< 3000 μm	33	7	16	10	28 (84.8)	5 (15.2)	1		
Submucosal invasion thickness									
≥ 2000 μm	94	3	8	83	62 (66.0)	32 (34.0)	2.025	0.963–4.259	0.063
< 2000 μm	64	12	28	24	51 (79.7)	13 (20.3)	1		

^a^*ESCC* esophageal squamous cell carcinoma

^b^*HE-VI* vascular invasion detected by hematoxylin and eosin (HE) staining

^c^*HE–LI* lymphatic vessel invasion detected by HE staining

^d^*IHC* immunohistochemistry

^e^*E-VI* vascular invasion detected by elastin staining + CK (AE1/AE3) immunohistochemistry

^f^*D-IHC* double immunohistochemistry

^g^*CD31-VI* vascular invasion detected by CD31 + CK (AE1/AE3) D-IHC

^h^*D2-40-LI* lymphatic vessel invasion detected by D2-40 + CK (AE1/AE3) D-IHC

^i^*L0, L1, and L2* the count of D2-40-LI was 0, 1–4, and 5 or more

^j^sm1, upper third of the submucosa; sm2, middle third of the submucosa; sm3, lower third of the submucosa

**Table 2:** Multiple logistic regression model for lymph node metastasis (LNM) in 158 T1b ESCC ^a^ patients

Clinicopathological characteristics	Odds ratio	95% CI	*P*
D2-40-LI ^b^			
L0 ^c^	1		
L1	1.061	0.443–2.544	0.894
L2	9.023	3.026–26.899	< 0.001
Degree of differentiation			
Well	1		
Moderate	3.496	0.883–9.475	0.060
Poor	4.492	1.162–17.371	0.029
Basaloid	4.519	0.917–22.263	0.064
Sarcomatoid	7.598	1.153–50.078	0.035

^a^*ESCC* esophageal squamous cell carcinoma

^b^*D2-40-LI *lymphatic vessel invasion detected by D2-40 + CK (AE1/AE3) double immunohistochemistry

^c^*L0, L1, and L2 *the count of D2-40-LI was 0, 1–4, and 5 or more

Four of 158 patients with pT1b ESCC were lost to follow-up. The follow-up period ranged from 3 to 256 months, with a median time of 68 months. Sixty-two patients (40.3%) experienced recurrence between 1 and 178 months, with a median recurrence time of 29.5 months. Nineteen patients (12.3%) developed distant metastasis between 1 and 192 months, with a median time of 31 months. The lung was the most common site of distant metastasis, accounting for 36.8% of cases.

**Fig. 3:**
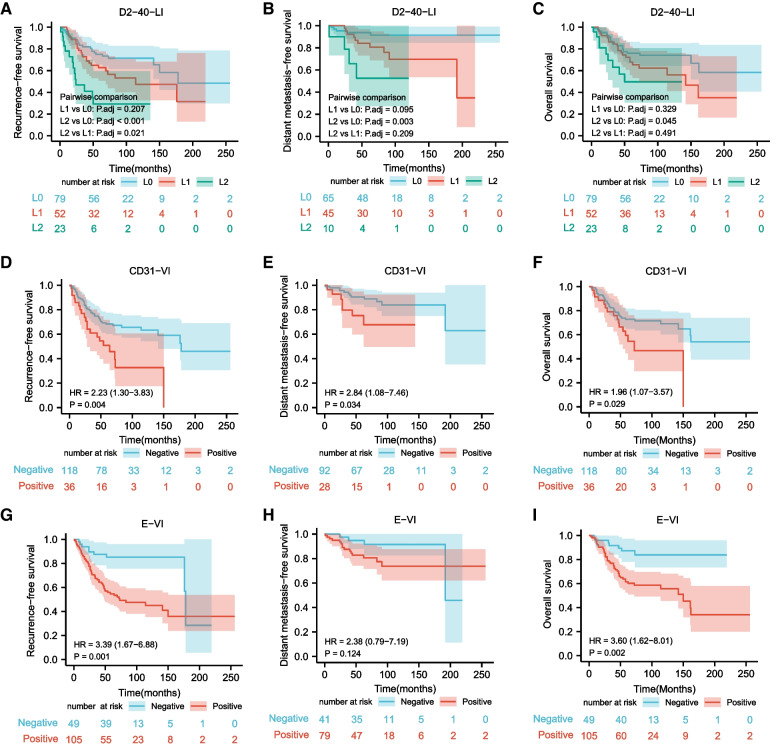
The survival curves of 154 patients with pT1b esophageal squamous cell carcinoma **A** Recurrence-free survival curves stratified by the count of lymphatic vessel invasion detected by D2-40 + CK (AE1/AE3) double immunohistochemistry (D2-40-LI). **B** Distant metastasis-free survival curves stratified by the count of D2-40-LI. **C** Overall survival curve stratified by the count of D2-40-LI. **D** Recurrence-free survival curves stratified by vascular invasion detected by CD31 + CK (AE1/AE3) double immunohistochemistry (CD31-VI). **E** Distant metastasis-free survival curves stratified by CD31-VI. **F** Overall survival curves stratified by CD31-VI. **G** Recurrence-free survival curves stratified by vascular invasion detected by elastin staining + CK (AE1/AE3) immunohistochemistry (E-VI). **H** Distant metastasis-free survival curves stratified by E-VI. **I** Overall survival curves stratified by E-VI

HE–LI, E-VI, CD31-VI, D2-40-LI, L2, tumor location (upper thoracic), and LNM were significantly associated with RFS and OS (*P* < 0.05; Table 3). HE–LI, CD31-VI, D2-40-LI, L1, L2, and LNM were significantly associated with DMFS (*P* < 0.05; Table 3). The RFS, DMFS, and OS curves of 154 patients classified by D2-40-LI, CD31-VI, and E-VI are shown in Fig. 3. Multivariate Cox regression analysis showed that E-VI, CD31-VI, L2, and tumor location (upper thoracic) were independent prognostic factors of RFS, with L2 being the most important prognostic factor (HR = 4.609; Table 4). E-VI, tumor location (upper thoracic), and LNM were independent adverse prognostic factors of OS, with E-VI being the most important factor(HR = 2.908; Table 4). LNM was an independent adverse prognostic factor for DMFS (HR = 16.187, 95% CI: 6.211–42.183; *P* < 0.001).

**Table 3:** Relationship between the clinicopathological characteristics and recurrence-free survival (RFS), overall survival (OS) and distant metastasis-free survival (DMFS) of 154 pT1b ESCC ^a^ patients

Clinicopathological characteristics	Total	Recurrence (%)	Distant metastasis (%)	Univariate Cox proportional hazards analysis								
				RFS			OS			DMFS		
				HR	95% CI	*P*	HR	95% CI	*P*	HR	95% CI	*P*
Lymphovascular invasion												
HE-VI ^b^ +	6	4 (66.7)	0 (0)	1.947	0.704–5.382	0.199	1.002	0.243–4.135	0.998	0.047	0.000–9023.544	0.623
HE-VI-	148	58 (39.2)	19 (12.8)	1			1			1		
HE–LI ^c^ +	33	19 (57.6)	8(24.2)	2.361	1.369–4.072	0.002	2.251	1.243–4.076	0.007	4.539	1.782–11.563	0.002
HE–LI-	121	43 (35.5)	11 (9.1)	1			1			1		
E-VI ^d^ +	105	53 (50.5)	15(14.3)	3.350	1.651–6.798	0.001	3.553	1.599–7.897	0.002	2.379	0.788–7.185	0.124
E-VI-	49	9 (18.4)	4(8.2)	1			1			1		
Peritumoral E-VI +	60	31 (51.7)	7(11.7)	1.611	0.979–2.652	0.061	1.654	0.836–3.275	0.149	0.950	0.374–2.418	0.915
Peritumoral E-VI-	94	31 (33.0)	12(12.8)	1			1			1		
CD31-VI ^e^ +	36	20 (55.6)	7(19.4)	2.226	1.296–3.824	0.004	1.925	1.069–3.564	0.029	2.838	1.082–7.442	0.034
CD31-VI-	118	42 (35.6)	12(10.2)	1			1			1		
Peritumoral CD31-VI +	28	15 (53.6)	4(14.3)	2.079	1.151–3.751	0.015	1.586	0.806–3.121	0.182	1.702	0.554–5.227	0.353
Peritumoral CD31-VI-	126	47 (37.3)	15(11.9)	1			1			1		
D2-40-LI ^f^ +	75	38 (50.7)	14(18.7)	2.109	1.262–3.524	0.004	1.836	1.049–3.214	0.033	3.727	1.341–10.361	0.012
D2-40-LI-	79	24 (30.4)	5(6.3)	1			1			1		
Peritumoral D2-40-LI +	64	29 (45.3)	9(14.1)	1.428	0.867–2.355	0.162	1.119	0.640–1.956	0.693	1.562	0.633–3.853	0.333
Peritumoral D2-40-LI-	90	33 (36.7)	10(11.1)	1			1			1		
L0 ^g^	79	26 (30.4)	5(6.3)	1			1			1		
L1	52	22 (46.2)	10(19.2)	1.668	0.946–2.942	0.077	1.624	0.886–2.978	0.117	3.064	1.045–8.980	0.041
L2	23	14 (60.9)	4(17.4)	4.135	2.111–8.101	< 0.001	2.769	1.253–6.118	0.012	8.093	2.152–30.441	0.002
Age												
≥ 60	58	25 (34.1)	7(12.1)	1.034	0.622–1.791	0.897	1.216	0.698–2.118	0.491	1.040	0.403–2.682	0.936
< 60	96	37 (38.5)	12(12.5)	1			1			1		
Sex												
Male	111	45 (40.5)	17(15.3)	1			1			1		
Female	43	17 (39.5)	2(4.7)	1.141	0.648–2.088	0.649	1.271	0.689–2.343	0.443	0.319	0.073–1.388	0.128
Degree of differentiation												
Well	30	11 (36.7)	1(3.3)	1			1			1		
Moderate	59	23 (39.0)	8(13.6)	1.127	0.549–2.314	0.745	1.067	0.479–2.378	0.873	4.743	0.587–38.307	0.144
Poor	38	16 (42.1)	5(13.2)	1.225	0.568–2.641	0.604	1.243	0.531–2.911	0.616	4.832	0.562–41.558	0.151
Basaloid	19	10 (52.6)	3(15.8)	1.538	0.653–3.642	0.325	1.668	0.661–4.205	0.278	7.998	0.825–77.522	0.073
Sarcomatoid	8	2 (25.0)	2(25.0)	0.747	0.165–3.375	0.705	0.941	0.202–4.370	0.938	8.773	0.794–96.872	0.076
LNM												
Yes	42	21 (50.0)	8(19.0)	1.769	1.044–2.997	0.034	1.976	1.111–3.514	0.020	16.187	6.211–42.183	< 0.001
No	112	41 (36.6)	11(9.8)	1						1		
Tumor location												
Upper thoracic	30	20 (66.7)	5(16.7)	2.738	1.567–4.785	< 0.001	2.659	1.440–4.911	0.002	2.306	0.767–6.938	0.137
Middle thoracic	100	33 (33)	9(9.0)	1			1			1		
Lower thoracic	24	9 (37.5)	5(20.8)	1.173	0.561–2.454	0.671	1.249	0.565–2.762	0.583	2.600	0.869–7.780	0.087
Macroscopic type												
Plaque-like	81	34 (42.0)	7(8.6)	1			1			1		
papillary	18	3 (16.7)	1(5.6)	0.333	0.102–1.083	0.068	0.253	0.060–1.061	0.060	0.488	0.060–3.999	0.504
Erosive	31	11 (35.5)	6(19.4)	0.801	0.406–1.584	0.524	0.726	0.343–1.535	0.402	1.913	0.641–5.707	0.245
Ulcerative	9	7 (77.8)	2(22.2)	2.337	1.027–5.341	0.043	2.077	0.854–5.049	0.107	4.081	0.827–20.138	0.084
Intraluminal mass	15	7 (46.7)	3(20.0)	1.770	0.778–4.027	0.174	1.382	0.532–3.593	0.506	3.710	0.943–14.600	0.061
Depth of tumor invasion ^h^												
sm1	15	3 (20%)	0(0)	1			1			1		
sm2	36	15 (41.7%)	5(13.9)	2.321	0.671–8.026	0.183	2.595	0.581–11.599	0.212	-	-	0.928
sm3	103	44 (42.7%)	14(13.6)	2.645	0.821–8.524	0.103	3.222	0.776–17.373	0.107	-	-	0.926
Tumor thickness												
≥ 3000 μm	121	50 (41.3)	13(10.7)	1.448	0.769–2.729	0.252	1.419	0.709–2.843	0.323	0.820	0.309–2.172	0.689
< 3000 μm	33	12 (36.4)	6(18.2)	1			1			1		
Submucosal invasion thickness												
≥ 2000 μm	90	38 (42.2)	11(12.2)	1.493	0.890–2.504	0.129	1.762	0.984–3.154	0.057	1.591	0.615–4.114	0.339
< 2000 μm	64	24 (37.5)	8(12.5)	1			1			1		

^a^*ESCC* esophageal squamous cell carcinoma

^b^*HE-VI*: vascular invasion detected by hematoxylin and eosin (HE) staining

^c^*HE–LI* lymphatic vessel invasion detected by HE staining

^d^*E-VI* vascular invasion detected by elastin staining + CK (AE1/AE3) immunohistochemistry

^e^*CD31-VI* vascular invasion detected by CD31 + CK (AE1/AE3) double immunohistochemistry

^f^*D2-40-LI* lymphatic vessel invasion detected by D2-40 + CK (AE1/AE3) double immunohistochemistry

^g^*L0, L1, and L2 *the count of D2-40-LI was 0, 1–4, and 5 or more

^h^sm1, upper third of the submucosa; sm2, middle third of the submucosa; sm3, lower third of the submucosa

**Table 4:** Multivariate Cox proportional hazards models for recurrence-free survival (RFS) and overall survival (OS) in 154 pT1b ESCC ^a^ patients

Clinicopathological characteristics	RFS			OS		
	HR	95% CI	*P*	HR	95% CI	*P*
Tumor location						
Upper thoracic	2.598	1.447–4.668	0.001	2.419	1.292–4.530	0.006
Middle thoracic	1			1		
Lower thoracic	1.305	0.617–2.759	0.486	1.196	0.537–2.663	0.661
E-VI ^b^						
Positive	2.402	1.160–4.974	0.018	2.908	1.288–6.568	0.010
Negative	1			1		
CD31-VI ^c^						
Positive	2.832	1.594–5.034	< 0.001	-	-	-
Negative	1			-	-	-
D2-40-LI ^d^						
L0	1			-	-	-
L1	1.327	0.737–2.389	0.345	-	-	-
L2	4.609	2.273–9.344	< 0.001	-	-	-
LNM ^e^						
Yes	-	-	-	1.983	1.096–3.587	0.024
No	-	-	-	1		

^a^*ESCC* esophageal squamous cell carcinoma

^b^*E-VI* vascular invasion detected by elastin staining + CK (AE1/AE3) immunohistochemistry

^**c**^*CD31-VI* vascular invasion detected by CD31 + CK (AE1/AE3) double immunohistochemistry

^d^*D2-40-LI* lymphatic vessel invasion detected by D2-40 + CK (AE1/AE3) double immunohistochemistry. L0, L1, and L2: the count of D2-40-LI was 0, 1–4, and 5 or more

^e^*LNM* lymph node metastasis

In addition, we evaluated the count of D2-40-LI of one representative paraffin block and conducted a relevant analysis. The trend was the same as in the analysis results of all paraffin blocks containing infiltrating carcinoma. However, the predicted value for LNM of one representative paraffin block (OR: 5.444) was slightly lower than that of all paraffin blocks containing infiltrating carcinoma (OR: 6.889) (Table S1 and Figure S1).

VI and LI were classified as intratumoral or peritumoral based on their location. As shown in Table 5, intratumoral-positive cases alone were the most common among all E-VI-positive patients (47/109), while peritumoral-positive cases alone were the most common among all CD31-VI- and D2-40-LI-positive cases (22/40, 45/78). However, no significant difference was found in VI/LI with different locations, either for LNM or RFS.

**Table 5:** Lymph node metastasis (LNM) and recurrence of pT1b ESCC patients with VI^a^ /LI^b^ in different locations (intratumoral or peritumoral region)

Type of VI/LI	Total	LNM (%)	*P*	Recurrence (%)	*P*
E-VI^c^ +	109	35 (32.1)		53 (48.6)	
Intratumoral region alone	47	14 (29.8)	0.129	22 (46.8)	0.538
Peritumoral region alone	22	11 (50.0)		13 (59.1)	
Both of two	40	10 (25.0)		18 (45.0)	
CD31-VI^d^ +	40	15 (37.5)		20 (50.0)	
Intratumoral region alone	8	4 (50.0)	0.357	5 (62.5)	0.311
Peritumoral region alone	22	9 (40.9)		12 (54.5)	
Both of two	10	2 (20.0)		3 (30.0)	
D2-40-LI^e^ +	78	27 (34.6)		38 (48.7)	
Intratumoral region alone	11	1 (9.10)	0.081	9 (81.8)	0.116
Peritumoral region alone	45	16 (35.6)		19 (42.2)	
Both of two	22	10 (45.5)		10 (45.5)	

^a^*VI* vascular invasion

^a^*LI* lymphatic vessel invasion

^c^*E-VI* vascular invasion detected by elastin staining + CK (AE1/AE3) immunohistochemistry

^d^*CD31-VI* vascular invasion detected by CD31 + CK (AE1/AE3) double immunohistochemistry

^e^*D2-40-LI* lymphatic vessel invasion detected by D2-40 + CK (AE1/AE3) double immunohistochemistry

## Discussion

Pathologists generally use separate immunohistochemical analyses of vasculature markers in daily work to identify LVI. However, it is difficult to determine whether the vessels contain tumor cells in some cases. In our study, adding epithelial markers made our study results more accurate. Using E&IHC and D-IHC for all blocks containing invasive carcinoma from each patient to evaluate LVI could better represent the true incidence of LVI. E&IHC and D-IHC significantly improved the detection rate of LVI and distinguished the type more accurately.

For VI detection, the sensitivity of HE staining was low (just 4.4%), and it is necessary to carry out E&IHC and D-IHC simultaneously. VI shown by D-IHC and E&IHC revealed different blood vessels. E-VI is characterized by thick-walled venous vessels with elastic fiber. Because of tumor invasion, no apparent lumen structure is observed, and vascular endothelial cells are destroyed; therefore, detection by HE staining or IHC is challenging. Additionally, VI sometimes resembles the spaces formed by cancer nest contraction with HE staining (Fig. 2 C). However, the structure of the vascular wall is shown clearly with E&IHC (Fig. 2 D). Most of the vascular types of VI displayed by CD31 D-IHC were capillaries, which were negative in E&IHC because of the lack of elastic fiber. Furthermore, capillaries were not easily distinguished from lymphatic vessels by HE staining, and CD31 D-IHC was also helpful in distinguishing the types of LVI, especially in the intratumoral region (Fig. 1 D, E). There was no significant correlation between HE-VI and LNM, RFS, or OS. However, both E-VI and CD31-VI were significantly associated with OS and were independent adverse prognostic factors for RFS. CD31-VI was significantly associated with distant metastasis (DM). Although there was no statistical correlation between E-VI and DM, the DMFS of E-VI-positive patients was poorer than that of E-VI-negative patients before a follow-up time of 150 months (Fig. 3 H). The lack of a significant difference may be due to the few cases of distant metastases.

In breast cancer, because few examples of intratumoral LVI without concomitant peritumoral emboli exist, Lee A K et al*.* mainly analyzed in detail the importance of peritumoral LVI [27]. The literature showed that artifactual spaces in the tumor resulting from the shrinkage of cell clusters are easily misdiagnosed as LVI in breast cancer, but misinterpretation can be avoided in the peritumoral region [33]. Thus, LVI must be diagnosed outside the border of invasive carcinoma in guidelines for breast cancer from the College of American Pathologists [34]. However, in our study on T1b ESCC, E&IHC and D-IHC could accurately identify LVI and prevent misdiagnosis. Furthermore, more intratumoral LVI-positive patients without peritumoral LVI were observed by D-IHC, and the rates of LNM and recurrence of these patients were not low (Table 5). Thus, intratumoral LVI should also be considered in T1b ESCC.

In the present study, both HE–LI and peritumoral D2-40-LI were significantly correlated with LNM. However, there was no significant correlation between D2-40-LI and LNM. We believe that the reason for the difference is due to the sensitivity of D-IHC to LI, which is significantly higher than that of HE staining, thereby greatly increasing the D2-40-LI positivity rate. Therefore, D2-40-LI was further grouped according to its count. L2 (the count of D2-40-LI was 5 or more) was significantly associated with LNM, RFS, DMFS, and OS, which had a larger Odds ratio and HR than HE–LI (Tables 1, and 3). L2 was also the strongest independent predictor for LNM and RFS in pT1b ESCC. Moriya H et al*.* divided LI into four groups according to the number of LI (0, 1–2, 3–9, and ≥ 10) in the IHC analyses [23]. They showed that the grade of D2-40-LI had a better predictive value for LNM, consistent with our results. However, the basis for grading was not clearly stated in their study. Further research is required to identify consistent cutoff points.

The literature suggests that ESCC patients with LVI have a poor prognosis. However, insufficient studies exist on the relationship between LVI and the prognosis of SESCC patients [9, 35]. Oguma J. et al*.* suggested that LVI was the only independent factor of disease-free survival in patients with sm2 and sm3 lymph node-negative SESCC, but LVI was not found to have a stronger prognostic impact than LNM [13]. In our study, patients were followed up for a long period of time, with a median follow-up duration of 68 months. The results showed that L2, E-VI, and CD31-VI were independent poor prognostic factors of RFS and OS. In particular, L2 (HR = 4.609) was the most potent adverse predictor of RFS (over LNM). Regarding the prognostic value of OS, the predictive value of E-VI also exceeded that of LNM (HR: 2.908 vs. 1.983).

The limitation of the study was that it was retrospective, and all patients had undergone thoraco-abdominal lymphadenectomy. Patients with sm3 accounted for a large proportion (107/158), and a certain degree of bias existed in the correlation analysis of the depth of invasion.

In conclusion, E-VI and CD31-VI were associated with DM and a poor prognosis. The D2-40-LI count is more important than the location, and high-frequency D2-40-LI has a better predictive value for LNM and RFS. In terms of the count of D2-40-LI, it is not necessary to analyze all paraffin blocks of invasive carcinoma if the pathologist can determine that the case belongs to the high-grade group (the count of D2-40-LI was 5 or more). We recommend using elastin staining and CD31/D2-40 IHC to identify LVI in all patients with pT1b ESCC based on the unoptimistic LNM rates and recurrence rates. Although E&IHC and D-IHC are more expensive and complicated, they are recommended if feasible due to their more clarity.

## Supplementary Information

**Additional file 1:** **Table S1. **Relationshipbetween the D2-40-LI of one representative paraffin block (RD2-40-LI) and lymphnode metastasis (LNM), recurrence-free survival (RFS), overall survival (OS)and distant metastasis-free survival (DMFS) of pT1b ESCC patients.

**Additional file 2:** **Figure S1. **The survival curves of 154 patients with pT1b esophageal squamous cellcarcinoma stratified by the count of D2-40-LI of one representative paraffinblock (RD2-40-LI).

## Data Availability

The datasets used and/or analyzed during the current study are available from the corresponding author on reasonable request.

## Abbreviations

LVI: Lymphovascular invasion
LNM: Lymph node metastasis
ESCC: Esophageal squamous cell carcinoma
SESCC: Superficial esophageal squamous cell carcinoma
IHC: Immunohistochemistry
E&IHC: Elastin staining + CK (AE1/AE3) immunohistochemistry
D-IHC: Double immunohistochemistry
VI: Vascular invasion
LI: Lymphatic vessel invasion
HE: Hematoxylin and eosin
HE-VI: Vascular invasion detected by HE staining
HE–LI: Lymphatic vessel invasion detected by HE staining
E-VI: Vascular invasion detected by elastin staining + CK (AE1/AE3) immunohistochemistry
CD31-VI: Vascular invasion detected by CD31 + CK (AE1/AE3) D-IHC
D2-40-LI: Lymphatic vessel invasion detected by D2-40 + CK (AE1/AE3) D-IHC
L0, L1 and L2: The count of D2-40-LI was 0, 1–4 and 5 or more
RFS: Recurrence-free survival
OS: Overall survival
DM: Distant metastasis
