# Supplementary material for: Prognostic significance of lymphovascular invasion in patients with pT1b esophageal squamous cell carcinoma
Source: BMC Cancer. 2023 Apr 22;23:370. doi: 10.1186/s12885-023-10858-7 (PMC10122816; doi:10.1186/s12885-023-10858-7)
Supplement: Supplementary file 1 — Additional file 1: Table S1. Relationshipbetween the D2-40-LI of one representative paraffin block (RD2-40-LI) and lymphnode metastasis (LNM), recurrence-free survival (RFS), overall survival (OS)and distant metastasis-free survival (DMFS) of pT1b ESCC patients. [file 12885_2023_10858_MOESM1_ESM.docx]

Table S1. Relationship between the D2-40-LI ^a^ of one representative paraffin block (RD2-40-LI) and lymph node metastasis (LNM), recurrence-free survival (RFS), overall survival (OS) and distant metastasis-free survival (DMFS) of pT1b ESCC ^b^ patients

| Clinicopathological characteristics | | Univariate logistic regression | | | | | Univariate Cox proportional hazards analysis | | | | | | | | | |  |  |
| --- | --- | --- | --- | --- | --- | --- | --- | --- | --- | --- | --- | --- | --- | --- | --- | --- | --- | --- |
|  |  | LNM | | | | | RFS | | | | OS | | | DMFS | | |  |  |
|  |  | OR | 95% CI | | | *P* | HR | | 95% CI | *P* | HR | 95% CI | *P* | HR | 95% CI | *P* |  |  |
| RD2-40-LI + | 1.890 | | | 0.936-3.815 | 0.076 | | 2.007 | 1.205-3.342 | | 0.007 | 1.718 | 0.985-2.997 | 0.057 | 3.768 | 1.356-10.471 | 0.011 |  | 1.049–3.214 |
| RD2-40-LI- | | 1 |  | | |  | 1 | |  | 1 |  |  | 1 |  |  |  |  |  |
|  | |  |  | | |  |  | |  |  |  |  |  |  |  |  |  |  |
| RL0 ^c^ | | 1 |  | | |  | 1 | |  |  | 1 |  |  | 1 |  |  |  |  |
| RL1 | | 1.110 | 0.491-2.506 | | | 0.802 | 1.543 | | 0.875-2.722 | 0.134 | 1.409 | 0.762-2.606 | 0.274 | 3.076 | 1.050-9.015 | 0.041 |  |  |
| RL2 | | 5.444 | 2.208-14.619 | | | 0.001 | 4.155 | | 2.219-8.110 | <0.001 | 3.032 | 1.417-6.489 | 0.004 | 8.617 | 2.266-32.773 | 0.002 |  |  |

^a^ D2-40-LI: lymphatic vessel invasion detected by D2-40+CK (AE1/AE3) double immunohistochemistry.

^b^ ESCC: esophageal squamous cell carcinoma.

^c^ The optimal cutoff point of the RD2-40-LI count for predicting LNM was 2.5 (area under the curve=0.626, and Youden index=0.231). According to the count, RD2-40-LI was subdivided into three groups: L0 (0), L1 (1-3), and L2 (≥3).
