# Supplementary figures and images for: Prognostic significance of lymphovascular invasion in patients with pT1b esophageal squamous cell carcinoma
Source: BMC Cancer. 2023 Apr 22;23:370. doi: 10.1186/s12885-023-10858-7 (PMC10122816; doi:10.1186/s12885-023-10858-7)

## Slide 1
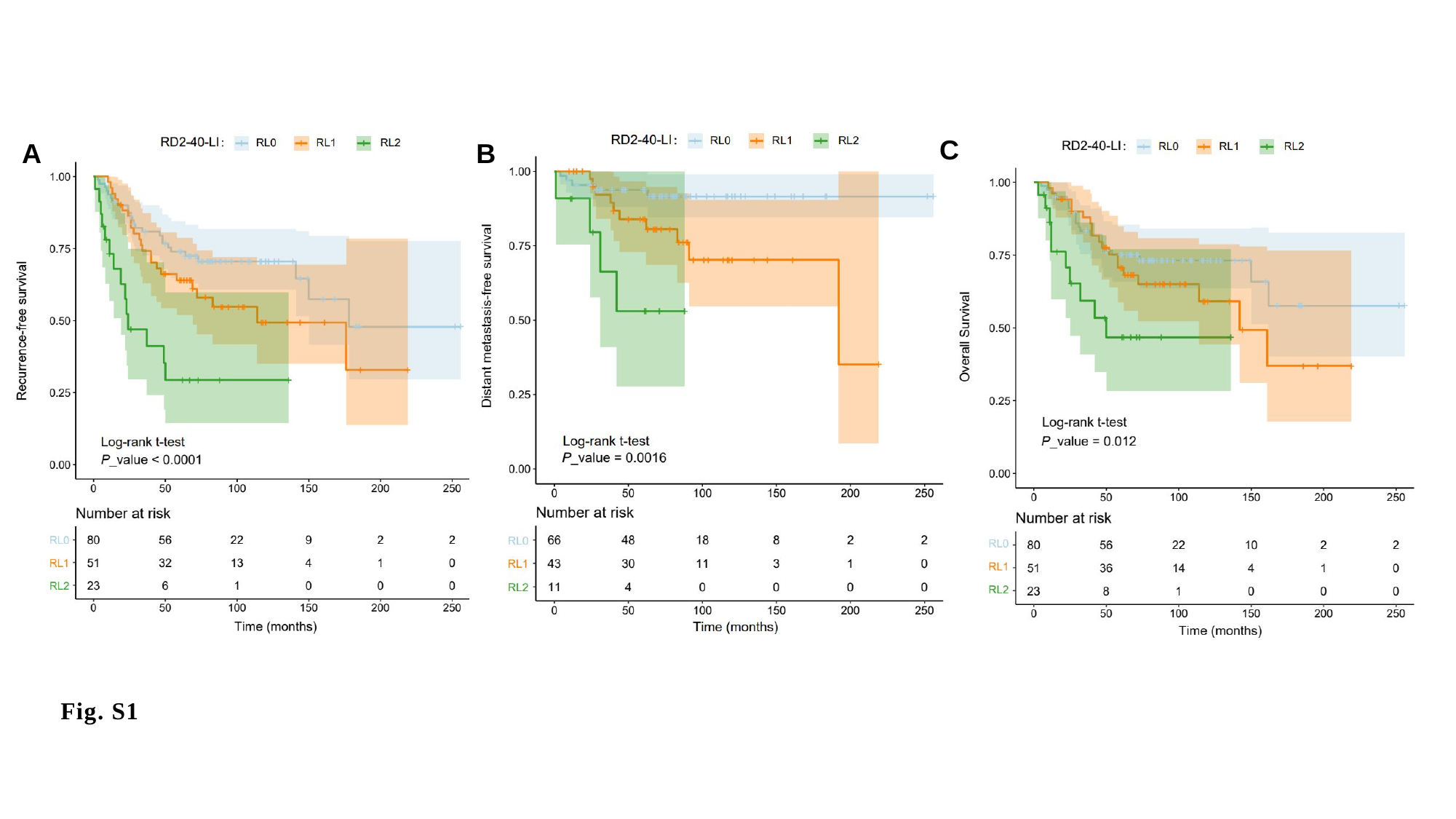

C
A
B
Fig. S1

Supplement: Supplementary file 2 — Additional file 2: Figure S1. The survival curves of 154 patients with pT1b esophageal squamous cellcarcinoma stratified by the count of D2-40-LI of one representative paraffinblock (RD2-40-LI). [file 12885_2023_10858_MOESM2_ESM.pptx]
